# The underlying mechanism between compulsory citizenship behaviors and employee innovative work behaviors and knowledge sharing: A moderated mediation model

**DOI:** 10.3389/fpsyg.2023.1128499

**Published:** 2023-02-14

**Authors:** Rawan Abukhait, Mohammad Nisar Khattak, Nessrin Shaya, Usha Ramanathan

**Affiliations:** ^1^Department of Management, Ajman University, Ajman, United Arab Emirates; ^2^Department of Education, American University in the Emirates, Dubai, United Arab Emirates; ^3^Nottingham Business School, Nottingham Trent University, Nottingham, United Kingdom

**Keywords:** higher education, compulsory citizenship behavior, negative affectivity, leadership, innovative work behavior, knowledge sharing, academics

## Abstract

**Purpose:**

This paper draws on conservation of resources theory to advance the literature on extra-role performance behaviors among academics, particularly innovative work behaviors and knowledge sharing, through the lens of work stressors.

**Methods:**

We develop a moderated-mediated model based on multi-source, multi-timed, and multi-level data from a sample of 207 academics and 137 direct supervisors in five higher education institutions in the United Arab Emirates (UAE).

**Findings:**

Results show that academics’ compulsory citizenship behaviors positively influence negative affectivity, which, in turn, negatively impacts academics’ innovative work behavior and knowledge sharing. The detrimental effect of compulsory citizenship behaviors on negative affectivity is then positively moderated by passive leadership, which amplifies this relationship. The combined effect of compulsory citizenship behaviors and negative affectivity exerted on innovative work behavior and knowledge sharing are magnified amid the elevated presence of passive leadership, while gender does not significantly influence this association.

**Originality:**

This is a pioneering study in the context of UAE to look into the counterproductive impact of CCB on employee innovative work behaviors and knowledge sharing.

**Implications:**

Pertinent theoretical and managerial implications are discussed.

## 1. Introduction

COVID-19 has changed the entire landscape of the teaching and learning portfolio of several educational institutions around the globe. In the United Arab Emirates (UAE), the education landscape after the COVID-19 pandemic is vastly different from that of the past. The UAE recognizes that robust and future-oriented education systems are a vital prelude for developing prosperous economies while facing the pandemic or any unexpected event to gain resilience. Accordingly, the country has one of the region’s most established education ecosystems, and it continues to attract investors, providers, and students. Its scope and ambition are unabated (Jeffery and Hancock, 2021).

The UAE is quickly evolving into a robust and engaging knowledge-based economy that emphasizes the development of a first-rate education system based on the UAE Vision 2021 National Agenda. The latter implies a complete transformation of the current education system and teaching methods and aims for all universities and students to be equipped with smart systems and devices as a basis for all instruction, assessments, and research (Vision, 2021). As a result, the UAE’s academic research output has surged 16-fold, with signs of abundant growth as of 2015, surpassing all expectations amid increased spending on research and development (Bardsley, 2020). Recently, The Times Higher Education World University Ranking 2020 has ranked a leading university in the UAE as the top 301st worldwide. Similarly, another global ranking, the QS World University Rankings, named at least six top-ranked UAE institutions. As a result, UAE continues to be the most competitive country in the Arab World according to the Global Competitiveness Index (Jeffery and Hancock, 2021), and education is well distinguished as a fundamental element of the country’s development (Vision, 2021) and a key driver of competitiveness. Yet, that may also imply that academics are prone to more stress than other vocations, and the morphosis of an academic career is a high-intensity stressor.

Given the potentially harmful influence of elevated occupational stress in causing extreme psychological reactions (Bachem et al., 2020), socio-emotional stress on faculty (Sinclair et al., 2020), and other associated counterproductive work-related outcomes, a further research discussion of factors impacting the education sector will be very appropriate in the current post-pandemic context. This study aims to advance the literature on extra-role performance behaviors among academics to provide recommendations toward sustaining high-quality faculty outputs against modern occupational stressors that negatively impact employee wellbeing to the detriment of performance. Given that, this study strives to answer an overarching research questions of “to what extent compulsory citizenship behaviors elicits negative emotions which in turn attenuate employee innovative work behaviors and their knowledge sharing?” Furthermore, this study also tends to explore that “whether passive leadership and gender work as boundary conditions in relationship between compulsory citizenship behaviors and employee negative affectivity?”

## 2. Theoretical background

To understand the various stress factors, we have searched the existing literature and identified important work hindrance pressures that can lead to an academic’s counterproductive work behavior. This section presents our literature findings in a succinct way under five dimensions: compulsory citizenship behavior (CCB), negative affectivity (NA), innovative work behavior (IWB), knowledge sharing (KNS), and passive leadership (PL).

Organ (1988) coined the term “organizational citizenship behavior” (OCB), defined as individual behavior that is discretionary, not explicitly acknowledged by the organization’s formal reward, and that, as a whole, improves the organization’s performance and efficiency (He et al., 2019). As a result, businesses are trying to determine how to achieve a high level of citizenship behaviors (CBs) (He et al., 2019). However, Vigoda-Gadot (2006) established that a unique aspect of CBs or extra-role activities–one that is less voluntary but nevertheless reflects extra effort at work–is referred to as “compulsory citizenship behavior” (He et al., 2019).

Although prior research has offered many insights into CB (such as Bolino and Turnley, 2005; Zhao et al., 2013; Hofmann and Stokburger-Sauer, 2017), the existence of such insights and their contributions to the educational organization context have been overlooked, and relevant studies could not be identified in the literature (Donglong et al., 2020). One reason is that institutional service is difficult to define; another is that academic staff place a lower value on service as opposed to teaching, research, and other academic duties (Ward, 2003).

At present, universities worldwide share the struggle to remain competitive, which is even more concerning considering the declining rate of high school graduates. In addition to their in-role behavior, an academic’s spontaneous manifestation of extra-role behavior has become a major force that drives faculty to show more CBs. As a result, we decided to focus our research on CCB’s dark and deterring side. We demonstrate this with a higher education faculty and investigate the implications for CCB, notably IWB and KNS. To support this research, we collected data using a survey questionnaire. We used a few variables to measure this construct, namely, social pressure on employees and multiple workplace tasks beyond employees’ usual capacity (He et al., 2020).

Universities play a key role in advancing innovative performance, which is a critical component of innovation to boost economic growth (Salem, 2014). It is through knowledge and innovation that institutions can support long-term survival and achieve sustained competitive advantage and economic repercussions (Antwi et al., 2019; Hermida et al., 2019). It is also well known that academics significantly contribute to the conceptualization and development of new knowledge, ideas, models, practices, technologies, tools, and methodologies. Sharing knowledge with colleagues allows academics to interact, exchange, and deliberate ideas with peers, direct their attention to the merit of ideas and turn ideas into viable solutions (Mura et al., 2013). Only *via* IWB and KNS can these advancements be realized. Researchers have long identified job stressors as having critical impacts on extra-role performance, such as employee innovation and creativity (Janssen, 2000; He et al., 2019). A recent study found that knowledge hiding by supervisor from subordinates enhance their subordinates’ disengagement which in turn transform into their reduced supervisor directed citizenship behaviors and enhanced supervisor directed silence (Arain et al., 2021). A study also found that formal and informal knowledge sharing results into higher performance in manufacturing firms (Wen and Wang, 2022). However, little is known about how and when CCB might exert a negative influence on IWB and KNS. In principle, job stress results in counterproductive responses, which are reflected in absenteeism, staff turnover, and reduced behavioral performance. However, the literature also reports that this relationship is not always necessarily negative. The challenge–hindrance pressures framework suggests that different stressors affect employees in different ways, where employees may respond innovatively to cope with changes in job nature or organizational settings (He et al., 2020). Recent meta-analytic findings identified the differential effect of knowledge hiding and knowledge sharing, specifically explored that employee turnover intention results into actual turnover in those organizations which are highly related to knowledge hiding than knowledge sharing (Arain et al., 2022a). This study further confirmed that the impact of knowledge hiding is more substantial on negative outcomes such as distrust and turnover intentions than on positive outcomes such as job satisfaction, task performance, extra-role performance and innovative performance. Additionally, while a large number of researchers have directed attention to inhibitors to KNS among employees and its antecedents (Muller et al., 2005; Michailova and Hutchings, 2006; Magnier-Watanabe and Senoo, 2010), little research has focused on job stressors as a key antecedent, and, to date, no studies have attempted to understand the said relationship within the higher education context. Research further supports that KNS continues to be an area that is under-researched compared to other organizational factors, despite its powerful impact in the workplace concerning more effective and productive teams and individual performance.

Consequently, drawing upon the abovementioned findings, we concentrate unambiguously on the outcomes of CCB for academics’ IWB and KNS. On the one hand, academics’ discontent with their work as a result of increased CCB will avert them from engaging in developing novel ideas and suppress KNS in favor of withholding knowledge. Because of those extra-role tasks against desire (Vigoda-Gadot, 2007), individuals have to devote considerable time, effort, and psychological resources to thrive; thereby, the motive to engage in innovativeness and share knowledge will dwindle. The underlying mental and emotional exhaustion can further suppress their innate innovative abilities (Hobfoll, 2012) and internal contextualization of knowledge transfer and sharing motivation (Mohsin et al., 2022).

Based on the above arguments, we aim to advance the literature on extra-role performance behaviors among academics, particularly IWB and KNS, through the lens of work stressors. We consider the following research questions for this study:

(A) What are the underlying factors of stress in the workplace that can dilute academics’ innovativeness and inhibit KNS behaviors?
(B) What are the potential processes responsible for the CCB–IWB and KNS relationship?
(C) How can education institutions sustain a high-quality faculty output against modern occupational stressors that negatively impact employee wellbeing to the detriment of performance?

On the other hand, prior research has also confirmed that an individual’s affect state–representing the personal perception of external social factors–is the proximal antecedent of work attitudes (e.g., productivity, job satisfaction, etc.) (Weiss and Cropanzano, 1996) that can promote productivity and inspire spontaneous work involvement and engagement (Lee et al., 2021). However, CCB is likely to daunt positive affect states, triggering perceived threats to the faculty’s goal of high self-esteem and the goal of living by justice as a result of increased psychological stress and reduced psychological wellbeing. This could ultimately lead to heightened feelings of negative affectivity and enthusiasm in behaving innovatively and sharing knowledge. The conservation of resources (COR) theory (Hobfoll, 1989) provides further support for the former implications, where CCB implies that academics have to invest cognitive, emotional, and physical resources in extra-role behavior and informal tasks beyond job duties against their free will (Vigoda-Gadot, 2006). All these activities require a heightened level of energy consumption (i.e., time and psychological resources). Thus, fewer resources can be dedicated to formal job responsibilities (Liu et al., 2019), driving emotions of anguish and distress, which consequently affect performance outcomes. Although previous research has raised concerns about the personal or organizational costs of performing CCBs, studies examining mediational pathways in defining the link between CCBs and output variables are limited, and little is known about the CCB–NA association, which could have critical and direct impacts. This research gap highlights the necessity for more studies that examine the relative importance of essential affective and cognitive characteristics in explaining the relationship between CB and performance outputs.

Our research study aims to contribute to this growing area of research by focusing on the mediating role of NA, which explains the relationship between CCB and faculty IWB and KNS, with significant potential implications at both the theoretical and empirical levels. Utilizing COR theory (Tierney and Farmer, 2002), we discuss the mediating mechanism of CCB–IWB and KNS by examining the influence of NA as an affect state. For academics with a higher level of negative affect, CCB will drive the individual to respond more aggressively and anxiously and with problem-based responses. Our study further employs COR theory to examine the contextual condition of the CCB–NA–IWB and KNS relationship. In accordance, a lack of a proper social support system from leaders amid the presence of CCB would have a detrimental impact on academics’ performance. Prior research confirms that PL (i.e., a leader who is aloof, non-communicative, indifferent, and fails to provide feedback) exacerbates NA (Kelloway and Day, 2005). Passive leaders are prone to failing to intervene when problems arise (Barling and Frone, 2017), failing to define how to deal with such demands within the organization, and failing to take an active stance on what should be done. Furthermore, PL is marked by a lack of effective employee social support, resulting in a lack of social resources for employees to manage their formal job responsibilities (Kelloway et al., 2005).

Consequently, the present study develops a moderated-mediation model to investigate the CCB, IWB, and KNS association, as well as the underlying mechanism and contextual condition of this relationship. We aim to address the extent to which CCB influences academics, IWB, and KNS through the mediating role of NA and moderating effect of PL as a boundary condition.

The strength of this research lies in employing the combined effect of two constructs (CCB and PL) that have never been studied before in relation to NA and, as a result, performance outcomes proxied by IWB and KNS among academics in higher education institutions.

**FIGURE 1:**
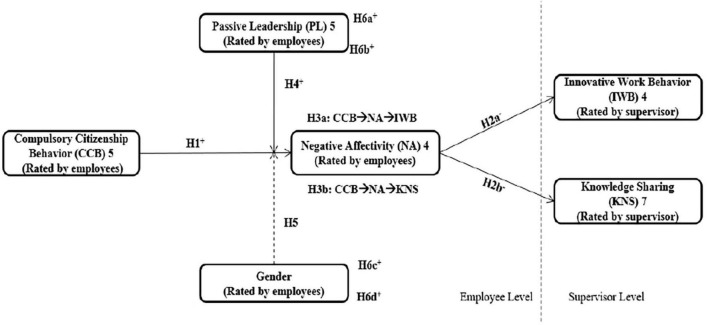
Conceptual framework.

In addition, although *occupational stress* can affect both men and *women*, stress appears to be differently experienced between genders (Sanchez-Franco et al., 2009; Riquelme and Rios, 2010). Women are often found to report more negative affect than men, especially sorrow, unhappiness, and apprehension (Fujita et al., 1991; Costa et al., 2001). In that sense, based on COR theory, women’s resources are consumed more than those of their male counterparts, and, hence, we anticipate less engagement in extra-role behaviors by women in an attempt to preserve their resources. Therefore, we attempt to illuminate possible gender differences upon experiencing excessive pressure and demands at the workplace and when responding to high CCB situations. As a result, the proposed moderated-mediated model in this study improves the field by addressing non-traditional ways in which the experience of CCB negatively influences occupational outputs and what may aggregate this impact. As a result, Figure 1 presents the hypothesized correlations in this study.

## 3. Development of the research framework and hypotheses

In summary, the current research aims to achieve the following four goals: (1) examine the impact of CCB on academics’ IWB and KNS as key performance outputs, (2) explore the role of NA as a linking mechanism between CCB and IWB and KNS, (3) identify the role of passive leadership in aggregating the negative effects of CCB on performance outcomes *via* NA, and (4) examine the moderating role of gender in the CCB–NA–IWB and KNS association.

This study benefits behavioral science in various ways. Firstly, it fills a gap in the academic research by investigating how CCB is experienced, responded to, and dealt with by academics. In an academic institution, education experts agree that academics and their actions are the key force in student satisfaction and their associated teaching/learning experience. The key tendency of for-profit academic institutions is to foster long-term survival and achieve sustainable competitive advantage and economic consequences (Eidizadeh et al., 2017; Gërguri-Rashiti et al., 2017)–all of which place pressure upon academics to perform at a high level and innovate (Lukić and Lazarević, 2018). Therefore, it is critical to understand the role of antecedents and consequences of affective states experienced by academics. Secondly, it is expected that investigating the impact of key stress variables on the performance outputs of academics in the higher education sector will allow human resource managers to take a more holistic approach to stress management, which will be critical in developing a strategic plan to address workplace stress.

Finally, this is the first study to look at the complex relationship between perceptions of CCB, NA, IWB, and KNS, as well as the moderating influences of leadership and gender. Therefore, while this study expands the theoretical boundaries in a multidisciplinary way that links organizational behavior, higher education, and psychology disciplines, it also derives recommendations that are applicable to countries in different regions.

### 3.1. Compulsory citizenship behaviors and negative affectivity

Citizenship behavior–once deemed always positive–has also been questioned by organizational behavior researchers over the last few years. These researchers have brought to light the exclusive discretionary nature of CB and highlighted its potential negative effect. Vigoda-Gadot (2007) defined CCB as “the exploitative and abusive tendency of supervisors and managements” (p. 377), which often creates role conflict and task ambiguity and leads to high job insecurity and perceived organizational injustice (Zhang et al., 2018). To support this argument, He et al. (2019) reported CCB as a kind of hindrance stressor in the workplace. Similarly, a study found that going the extra mile (i.e., helping behaviors toward coworkers/supervisors beyond one’s job description) at the workplace due to managerial pressure creates feelings of burnout, overload, and anxiety among employees (He et al., 2020). A handful of studies have endorsed this harmful impact of CB, finding that CCB negatively influences employee wellbeing (Somech and Bogler, 2019), burnout (Zhang et al., 2018), motivation (De Clercq et al., 2021), work–family conflict, and intention to quit (Youn et al., 2017; Banwo and Du, 2020).

Negative Affectivity has been examined as both a stable dispositional trait and an emotion state. The conceptualization of NA as a momentary and transient state of negative emotions across certain situations and time (Bohle and Tilley, 1993) in organizational research is particularly useful in capturing instant emotional reactions in response to effective experience. To exam in academics emotional reaction to CCB and its detrimental effect on performance, this research distinguished NA as a momentarily emotional response, referring to academics’ affect state at a given moment, such as feelings of disguise, anger or fear (Wong et al., 2006; Chen et al., 2021). Such a state has unambiguous consequences on individual’s enthusiasm and initiatives, even leading to emotional exhaustion and physical burnout, that are of great relevance on the functioning of organizations. Individuals having higher NA scores experience higher distress, higher discomfort, and dissatisfaction when they encounter different situations at different times (Watson and Clark, 1984). These individuals ruminate on their mistakes and dwell on their shortcomings, which makes them more chronic, and resultantly they always look at the dark side of life (Watson et al., 1999). The affective event theory (AET) framework (Weiss and Cropanzano, 1996) has widely been used to explore the relationships among workplace events, emotions, and discretionary behaviors. In the preceding paragraph, it is evident that CCB works as a hindrance because of having the impetus of social and managerial pressures, which remove the true discretionary nature of CB. Taking this as a potential hindrance stressor, CCB might create negative emotions, which may later transform into counterproductive behaviors. Hence, we postulate the following hypothesis:

H1. Compulsory citizenship behaviors will positively influence employees’ negative affectivity.

### 3.2. Negative affectivity and employee innovative work behaviors and knowledge sharing

Negative affectivity includes affective states like “anger, scorn, revulsion, guilt, self-dissatisfaction, a sense of rejection and sadness” (Watson and Clark, 1984, p. 465). Focusing on the potential association between NA and aggressive workplace behaviors, Geen and Donnerstein (1990) found that NA works as a predictor of workplace aggression. Berkowitz (1993) also confirmed that NA has a significant direct relationship with aggression. Furthermore, he describes those individuals having a higher NA score as more sensitive toward aversive outcomes and having a higher tendency to respond more aggressively toward negative stimulation compared to those who have a lower NA score. Another study by Bouckenooghe et al. (2013) found that positive and negative affectivity both have relationships with job performance and turnover intention. These authors further confirmed that this relationship is moderated by job satisfaction. A recent study found that knowledge hiding has a trickle-down effect where leader knowledge hiding persuade employee knowledge hiding which results into higher interpersonal deviance and reduced citizenship behaviors (Arain et al., 2022b).

High NA individuals are depressed and, most of the time, they are full of negative emotions, which induce them to exhibit a hostile attribution style; resultantly, they are more prone to aggressive behaviors in the workplace (Martinko and Zellars, 1998). Another study by Larsen and Ketelaar (1991) argues that NA strives to enhance the individual’s susceptibility toward those stimuli which generate outward-focused negative emotions like distress, anger, and anxiety. George (2000) reported that individuals high in NA view the world in a negative way and look at the environment as threatening to themselves. Similarly, s meta-analytic study by Spector and Jex (1998) found that NA positively predicts organizational constraints, workload anxiety, and interpersonal conflict. Studies have also confirmed a positive association between NA and role ambiguity, role conflict, and lack of autonomy (e.g., Dollard and Winefield, 1998; Fortunato et al., 1999; Zellars et al., 1999). Similarly, studies also report that NA leads to dysfunctional employee behaviors, including aggression (Balducci et al., 2012), counterproductive workplace behaviors (Spector and Fox, 2002; Samnani et al., 2014), and reduced performance (Seo and Ilies, 2009).

There is a dearth of literature on the relationship between NA and employee IWB and KNS. However, as described above, numerous studies have found that NA is detrimental to employee performance which, in turn, negatively influences the overall organizational performance. Personality literature confirms that the Big Five personality traits and proactive personalities positively predict employee KNS and IWB (De Vries et al., 2006; Belschak et al., 2010).

This study draws on COR theory (Hobfoll, 1989), which posits that individuals strive to gain and conserve the resources that they deem as valuable. The theory further argues that the threat of loss of resources and actual loss of these valuable resources both cause stress in individuals and, therefore, to avoid further resource losses, they will endeavor to restore their available resources (Hobfoll, 1989; Halbesleben et al., 2014). Studies have found that the presence of negative affect (e.g., anxiety and anger) might consume individuals’ resources by producing further cognitive and emotional demands (Beal et al., 2005; Karatepe and Tizabi, 2011). Therefore, employees who are high on NA are more likely to reduce their expenditure on physical and psychological resources and conserve them for their future use. In such a situation, they will be reluctant to share their valuable knowledge with others (colleagues and supervisors) and will also be unwilling to take the risk of doing something unusual. Hence, we propose the following hypotheses:

H2a. Negative affectivity has a negative effect on innovative work behavior.

H2b. Negative affectivity has a negative effect on knowledge sharing.

### 3.3. Mediating role of negative affectivity

We know from the counterproductive workplace behavior literature that counterproductive workplace behavior does not occur automatically. Past research has established the path from workplace stressors to counterproductive workplace behavior through an emotional process (Lee and Allen, 2002; Rodell and Judge, 2009). In line with this, past researchers have noted that CCBs act as workplace stressors and transform into employee silence *via* moral disengagement (He et al., 2019) and employee deviance and facades of conformity *via* emotional exhaustion (Liang et al., 2022).

The AET framework (Weiss and Cropanzano, 1996) best describes the relationship between workplace events, emotions, and subsequent behaviors. Proponents of AET support the notion that a lack of fairness in the workplace acts as a job stressor, which triggers negative emotions and, consequently, deviant workplace behaviors (Weiss and Cropanzano, 1996; Peng et al., 2016; Liang et al., 2022). Therefore, every event occurring at the workplace might produce emotional reactions among employees, which in turn transform into employee attitudinal and behavioral consequences (Liang et al., 2022). Taking this line of inquiry one step further, this study investigates whether CCB, employee IWB, and employees’ attitudes toward KNS have a distal relationship. NA, the most basic, discrete, negative emotion, works as an underlying mechanism between CCB, IWB, and KNS. According to the AET (Weiss and Cropanzano, 1996), when employees encounter CCB, negative emotions (NA) might arise, which are later transformed into IWB and KNS. Hence, we propose the following hypotheses:

H3a. Negative affectivity mediates the negative relationship between compulsory citizenship behavior and innovative work behavior.

H3b. Negative affectivity mediates the negative relationship between compulsory citizenship behavior and knowledge sharing.

### 3.4. Moderating role of passive leadership

Leadership researchers have mainly focused on the positive forms of leadership, such as transformational leadership (Bass and Avolio, 1991; Avolio et al., 1999), servant leadership (Newman et al., 2017), and ethical leadership (Kalshoven et al., 2013), and have paid little attention to the negative forms of leadership (e.g., passive leadership). However, in the recent past, leadership researchers have paid attention to the hazardous effects of negative leadership on individuals’ attitudinal and behavioral outcomes (Skogstad et al., 2007; Nauman et al., 2018). The most basic forms of negative leadership include laissez-faire and passive management by exception (Bass and Avolio, 1997; Judge and Piccolo, 2004). Laissez-faire is no leadership or the absence of leadership at a time of crisis, while passive management, by exception, is characterized by those who are reluctant to take any action until the problems become chronic (Kelloway et al., 2005; Holtz and Harold, 2013). These two dimensions of negative leadership are highly correlated; as such, leadership researchers have combined these into the single term of *passive leadership* (Den Hartog et al., 1997; Avolio et al., 1999).

Compulsory citizenship behavior has a negative connotation and a negative effect on an individual’s job satisfaction, job performance, and extra-role behavior but positively affects their job pressure and intention to leave the organization (Tepper et al., 2004; Vigoda-Gadot, 2007). It is also evident from the OCB literature that willingness to be a good citizen and go the extra mile for the organization always demands the investment of extra effort and energy in the workplace (Deery et al., 2017). In order to meet these inflated demands of job-related tasks and CBs, individuals spend more resources, which causes a drain on their resources and ultimately results in emotional strain (Halbesleben et al., 2009). Some OCBs demand a higher investment of resources; for example, helping colleagues at the workplace with their challenging job responsibilities can considerably increase an individual’s workload, taking time away from undertaking their own task responsibilities and depleting the resources needed to cope with family responsibilities (Marinova et al., 2010). Another demanding OCB type is conscientious behavior, where employees go the extra mile than their call of duty regarding work breaks, attendance, and preserving organizational rules, which might increase their workload (Deery et al., 2017). A study also found that employees who go beyond their minimum job responsibilities, such as coming early to the workplace and leaving late, are more likely to exhibit higher job stress and greater work–life conflict (Bolino et al., 2010).

There is ample evidence of the prevalence of PL in many organizations and its detrimental effects on workplace climate and social exchange relationship quality (Aasland et al., 2010; Lee, 2018). As described in the preceding paragraph, passive leaders are reluctant to take decisions at the time of crisis and, therefore, often fail to accomplish employee and organizational goals (Chênevert et al., 2015). Similarly, PL is negatively related to leadership effectiveness and follower satisfaction (Judge and Piccolo, 2004). While proposing that PL can exacerbate the positive effect of CCB on employee NA, we draw from the influential COR theory (Hobfoll, 1989). COR theory postulates that when encountering stress (e.g., due to loss/potential loss of resources, and/or lack of resource gain), individuals will invest existing resources in order to protect against the loss, potential loss, and/or lack of gain (Hobfoll, 1989). Therefore, we argue that in a highly demanding organization, employees will frequently face threats of resource loss and also face uncertainty about the likelihood of resource gain. Specifically, a culture characterized by self-serving, illegitimate, power-focused behavior will threaten a range of internal and external resources (e.g., reliable knowledge, peer support, job stability, etc.). We argue that passive leaders–who are known for their indifference and reluctance, specifically during a time of crisis (Kelloway et al., 2005; Harold and Holtz, 2015)–are likely to enhance employees’ negative emotions. Studies have also found that exhibiting PL behaviors by an immediate supervisor enhances employees’ work stress and interpersonal conflict (Skogstad et al., 2007). An organizational environment characterized by CCBs and PL might be highly challenging for employees to handle. In a highly demanding organization where employees are already under higher stress, the indifferent attitude of passive leaders will exacerbate this effect and heighten employees’ NA. Thus, we propose the following hypothesis:

H4: Passive leadership positively moderates the positive relationship between compulsory citizenship behavior and negative affectivity, such that the relationship is stronger when passive leadership is high.

### 3.5. Moderating role of gender

Previous studies note that women are often found to report more negative affect than men, especially sadness and anxiety (Fujita et al., 1991; Costa et al., 2001). Similarly, the prevalence of anxiety disorders (Reich, 1986) and depression (Nolen-Hoeksema, 2001) in women is almost double that of their male counterparts. These findings imply that men and women greatly differ in their sadness and anxiety. Managing stress and associated hurdles appear to be managed differently across genders when encountered with occupational stress due to excessive pressure and high demands in the workplace (Sanchez-Franco et al., 2009; Riquelme and Rios, 2010). In that sense, based on COR theory, women’s resources are consumed more and faster than those of their male counterparts, leading them to engage less in extra-role behaviors, particularly IWB and KNS, in an attempt to preserve their resources. Extending this line of inquiry, we argue that CCB is a kind of stressor that might trigger employees’ negative emotions; however, this impact will be stronger for women than men. Hence, we postulate the following:

H5: Gender moderates the positive relationship between compulsory citizenship behavior and negative affectivity.

### 3.6. Moderated-mediation effect

In developing H6 and H7, we argue that the positive effect of CCB on employee NA is moderated by PL and gender. Therefore, the mediated link between CCB and employee IWB and KNS can be regarded as a “first-stage moderated-mediation model” (Muller et al., 2005; Edwards and Lambert, 2007). In other words, the indirect effect of CCB on employee IWB and KNS is conditional on a high level of PL and gender. CCB should theoretically result in low IWB and KNS *via* employee NA; however, this effect is hypothesized to be inflated in the presence of PL and for women. Therefore, the mediating effect of NA on the relationship between CCB and employee IWB and KNS may vary according to whether there are high or low levels of PL and on a gender basis. Thus, the following hypotheses are proposed:

H6a: Passive leadership moderates the indirect effect of compulsory citizenship behavior on innovative work behavior through negative affectivity, such that the mediated effect is stronger when passive leadership is high.

H6b: Passive leadership moderates the indirect effect of compulsory citizenship behavior on knowledge sharing through negative affectivity, such that the mediated effect is stronger when passive leadership is high.

H6c: Gender moderates the indirect effect of compulsory citizenship behavior on innovative work behavior through negative affectivity, such that the mediated effect is stronger for females.

H6d: Gender moderates the indirect effect of compulsory citizenship behavior on knowledge sharing through negative affectivity, such that the mediated effect is stronger for females.

## 4. Materials and methods

### 4.1. Data collection and procedures

We used a survey questionnaire approach to collect data to answer our research questions (He et al., 2020; Mohsin et al., 2022). Data were collected in five large universities located in three emirates (i.e., Dubai, Abu Dhabi and Sharjah) in the UAE, out of which two were from Dubai, two from Abu Dhabi and one from Sharjah. Participants in this study were full-time faculty members and their direct supervisors. Respondents with full-time administrative appointments were excluded from the sample. Hence, this study focuses on faculty members engaged in teaching and related activities (including time in preparing courses, developing new curricula, advising or supervising students, supervising student internships and theses/dissertations, attending professional development activities), research/scholarship (including gathering and analyzing data; managing grants; preparing articles, chapter articles or books; attending or presenting at professional conferences; applying for external funding; participating in exhibitions related to fine or applied arts), and institutional and community service (including giving speeches).

As indicated earlier, job stressors have been identified as one of the critical and complex phenomena encountering higher education institutions. The typical “dreaming spires” perception of employment in higher education institutions is a rarefied, privileged, and high social standing, secure, low-stress, even cloistered existence of gowns, high table, and “tenure,” with the occasional leisurely seminars having long vanished (Poalses and Bezuidenhout, 2018).

A convenience sampling approach was employed to recruit subjects through the researchers’ professional and personal networks. Hence, institutions were selected based on accessibility and availability (Kucukusta et al., 2013). Online surveys were utilized to collect data, and surveys were shared with faculty members and their direct supervisors to improve the quality of the findings and reduce common method bias (Podsakoff et al., 2003). The participants were informed of the research purpose and the voluntary nature of participation, and a statement about confidentiality or anonymity was provided through the survey cover letter.

The administration of online surveys took place across two main phases. In Time I (September 2021), supervisors holding the role of Head of Departments, Department Chairs, and Program Directors were approached and invited to choose up to five faculty subordinates to participate in the study. Researchers developed a table with supervisors’ names and their subordinates’ contact details. In that sense, faculty participation was determined through the supervisors, followed by the researchers approaching the assigned subjects for participation consent. Each pair of supervisors and respective subordinates on the list was assigned and informed of their code and was requested to write the code in the dedicated slot on the survey. The coding stage allowed us to match the responses from the two sources and consolidate them for the subsequent analysis stage. In Time I, participating faculty members were invited to complete the survey and assess their supervisors and themselves on CCB, PL, and NA. In Time II (November 2021), participating supervisors were approached and requested to rate their subordinates’ KNS and IWB levels. Accordingly, a total of 137 supervisors participated, which is a true representation of the target population of academic supervisors. Responses that showed either a missing matching evaluation questionnaire from the supervisor side or the subordinate side were discarded. A total of 207 faculty members participated in the study resulting in matched responses. While each discipline was represented, most respondents were affiliated with Arts and Humanities, Business, and the fewest with Engineering. Among the participating employees, 57% were males and 43% were females; the majority (91%) were over 32 years of age, 38% had up to 10 years of experience, and 62% were more experienced.

### 4.2. Measurement development

Multiple items from highly reliable and well-established scales extracted from higher education and social sciences literature were used to measure all key variables. Among the individual characteristics are age, gender, current academic rank, years of employment at their current institution, and nationality.

Compulsory citizenship behavior (rated by faculty subordinates) was measured through a 5-item scale developed by Vigoda-Gadot, 2007. Sample items include, “The management in this organization puts pressure on employees to engage in extra-role work activities beyond their job tasks;” “There is social pressure in this organization to work extra hours, beyond the formal workload and without any formal rewards;” “I feel that I am expected to invest more effort in this job than I want to and beyond my formal job requirements;” “I feel that I am forced to help other teachers beyond my formal obligations and even when I am short on time or energy;” and “I feel that I am forced to assist my supervisor against my will and beyond my formal job obligations” [from *highly disagree* (1) to *highly agree* (5)].

Negative affectivity (rated by employees) was measured through a 4-item scale adopted from Wong et al. (2006). Sample items include, “My job makes me dissatisfied,” “My job makes me unhappy,” “My job makes me troubled,” and “My job makes me miserable” [from *strongly disagree* (1) to *strongly agree* (4)], that was also adopted by Chen et al., 2021).

Knowledge sharing (rated by supervisors) was measured through a 7-item scale adopted from Srivastava et al. (2006). Sample items include, “The subordinate shares his/her special knowledge and expertise with others;” “If the subordinate has some special knowledge about how to perform the task, he/she is likely to tell others about it;” “The subordinate exchanges information, knowledge, and sharing of skills with his/her coworkers;” “The subordinate freely provides other members with hard-to-find knowledge or specialized skills;” “The subordinate helps others in developing relevant strategies;” “The subordinate shares a lot of information with others;” and “The subordinate offers lots of suggestions to others” [from *highly disagree* (1) to *highly agree* (7)].

Innovative work behavior (rated by supervisors) was measured through a 4-item scale adopted from Welbourne et al. (1998). Sample items included: “He/she comes up with new ideas,” “He/she works to implement new ideas,” “He/she finds improved ways to do things,” and “He/she creates better processes and routines” [from *highly disagree* (1) to *highly agree* (4)].

Passive leadership (rated by employees) was measured through a 5-item scale adopted from Barling and Frone (2017). Sample items include, “My supervisor is unavailable when staff need help with a problem” and “My supervisor delays acting until problems become serious” [from *highly disagree* (1) to *highly agree* (5)].

### 4. 3. Control variables

In keeping with previous research, faculty subordinate age, experience, and nationality were controlled in this study. Supervisor age, gender, and years of experience, and nationality were also considered as control variables due to their likely influence on the study’s dependent variables (Luksyte et al., 2020).

## 5. Results

SPSS 26 was used to perform descriptive analysis on participants’ demographic characteristics and correlations among all the study variables. AMOS 20 was also used to run confirmatory factor analysis (CFA) to test the construct validity of all the measurements. In addition, this study performed Harman’s single-factor test to check whether common method variance was a serious problem, as this study employed a one-wave self-reported design, which means that the data of all the variables were collected at the same point in time. The findings of Harman’s single-factor test revealed that the one-factor model explained 42.073% of the total variance, suggesting that common method variance was not a serious problem (Hoyle, 1995). Given that each participant provided data at the supervisor level (i.e., IWB and KNS) and at the employee level (i.e., gender, CCB, PL, and NA), our hypotheses testing necessitated hierarchical or cross-level techniques. Since linear regression modeling is unable to resolve independence problems and estimate the impacts of factors at different levels simultaneously (Raudenbush and Bryk, 2002), we used hierarchical linear modeling (HLM) as an analytic tool to test our causal and moderation hypotheses (i.e., H1, H2a, H2b, H4, and H5). We relied on the recommendations of Preacher et al. (2007) and Hayes (2018) to test our moderated-mediation model by running two analyses. Specifically, we first utilized the PROCESS macro Model#4 developed by Hayes (2018) to test the mediation model (i.e., H3a and H3b). We then incorporated the moderator into the entire model (Edwards and Lambert, 2007; Preacher et al., 2007; Hayes, 2017), utilizing PROCESS macro Model#10, to test for the entire moderated-mediation model (i.e., H6a, H6b, H6c, and H6d). These models were tested using the bootstrapping technique with a 5,000 sample size (Hayes, 2017), which conducts a more reliable estimation of indirect effects and does not make assumptions about the normality of the sampling distribution, which are often unrealistic (Preacher and Hayes, 2008). Significant results were identified by examining the 95% confidence interval (CI) resulting from the bootstrapping mediation analyses. A CI not including zero indicates a significant mediation or moderation effect.

### 5.1. Confirmatory factor analyses findings

The CFA is assessed by two main components: convergence validity and discriminant validity. Table 1 represents the results of convergent validity, which refers to the degree to which multiple attempts to measure the same concept are in agreement (Hair et al., 2010).

**TABLE 1:** Convergent validity and internal reliability.

Construct	Item	Factor loading	Average variance extracted (AVE)	Composite reliability (CR)	Internal reliability Cronbach alpha
**Employee level (*n* = 207)**					
Compulsory citizenship behavior (CCB)	CCB1	0.883	0.742	0.935	0.935
	CCB2	0.86			
	CCB3	0.857			
	CCB4	0.842			
	CCB5	0.865			
Passive leadership (PL)	PL1	0.917	0.821	0.958	0.958
	PL2	0.928			
	PL3	0.91			
	PL4	0.869			
	PL5	0.906			
Negative affectivity (NA)	NA1	0.898	0.809	0.944	0.944
	NA2	0.887			
	NA3	0.9			
	NA4	0.913			
**Supervisor level (*n* = 137)**					
Innovative work behavior (IWB)	IWB1	0.92	0.807	0.944	0.943
	IWB2	0.894			
	IWB3	0.91			
	IWB4	0.869			
Knowledge sharing (KNS)	KNS1	0.863	0.813	0.968	0.968
	KNS2	0.911			
	KNS3	0.884			
	KNS4	0.896			
	KNS5	0.921			
	KNS6	0.913			
	KNS7	0.921			

As shown in Table 1, the results of assessing the standardized loadings of the items showed that the factor loading of all 25 items was more than 0.5, as recommended by Hair et al. (2006), ranging between 0.842 (for CCB4) and 0.928 (for PL2). The average variance extracted (AVE) of all the variables was above 0.5 and ranged between 0.742 (for CCB) and 0.821 (for PL). The composite reliability (CR) ranged between 0.935 (for CCB) and 0.968 (for KNS), which was higher than the suggested value of 0.6 (Hair et al., 2010). The Cronbach alpha values were more than 0.7, as recommended by Nunnally and Bernstein (1994), ranging between 0.935 (for CCB) and 0.968 (for KNS). These results indicate a satisfactory convergent validity.

Table 2 presents the means, standard deviations, correlations between constructs, and the results of discriminant validity, which refers to the issue of how truly distinct a construct is from other constructs (Fornell and Larcker, 1981; Hair et al., 2006).

**TABLE 2:** Discriminant validity, correlations, and descriptive statistics.

	Construct	Mean	SD	1	2	3	4	5	6
1	Gender_a_	1.430	0.496	(1)					
2	Compulsory citizenship behavior (CCB)	2.480	0.940	0.003	(0.862)				
3	Passive leadership (PL)	2.443	1.210	-0.024	0.222**	(0.906)			
4	Negative affectivity (NA)	2.354	1.101	0.125	0.500***	0.416***	(0.900)		
5	Innovative work behavior (IWB)	3.584	1.120	0.063	-0.123	-0.167*	-0.510***	(0.902)	
6	Knowledge sharing (KNS)	3.375	1.115	0.035	-0.220**	-0.243***	-0.583***	0.534***	(0.898)

Values in parentheses display the square root of the AVE; SD = standard deviation; All constructs have 5-point Likert scale: 1 = strongly disagree, 5 = strongly agree; a: gender has two groups: 1 = male, 2 = female; Standardized correlations reported *p < 0.05; **p < 0.01; and ***p < 0.001.

As shown in Table 2, the square root of the AVE for each construct is higher than the correlations of that construct with other constructs (Hair et al., 2010). Further, the correlations between constructs were all less than the threshold of 0.85, ranging between −0.584 (correlation between NA and KNS) and 0.534 (correlation between IWB and KNS), indicating a satisfactory discriminant validity between the constructs (Kline, 2010).

As Table 2 illustrates, CCB had a significant positive correlation with PL (*r* = 0.22, *p* < 0.01) and NA (*r* = 0.500, *p* < 0.001) but a significant negative correlation with KNS (*r* = −0.220, *p* < 0.01). PL had a significant positive correlation with NA (*r* = 0.416, *p* < 0.001) but a significant negative correlation with IWB (*r* = −0.167, *p* < 0.05) and KNS (*r* = −0.243, *p* < 0.001). NA had a significant negative correlation with IWB (*r* = −0.510, *p* < 0.001) and KNS (*r* = −0.538, *p* < 0.001). The correlation between KNS and IWB was found to be significant and positive (*r* = 0.534, *p* < 0.001).

Table 2 also provides the descriptive statistics of the constructs, including the mean and standard deviation. The lowest mean value belonged to NA (*M* = 2.354), while IWB had the highest mean value (*M* = 3.584). The lowest and highest standard deviation belonged to CCB (SD = 0.940) and PL (SD = 1.210), respectively.

The results unveiled a good fit between the hypothesized five-factor model and the data χ^2^(265) = 468.373 (*p* < 0.001); χ^2^/*df* = 1.767; the goodness-of fit-index (GFI) and adjusted goodness-of-fit index (AGFI) were 0.851 and 0.817, respectively, above the threshold of 0.8. The comparative fit index (CFI), Tucker-Lewis index (TLI), and incremental fit index (IFI) were 0.964, 0.959, and 0.964, respectively, all above the threshold of 0.9. The standardized root mean square residual (SRMR) and root mean square error of approximation (RMSEA) were 0.045 and 0.061, respectively. These model fit indices suggested a good fit of this measurement model.

### 5.2. Hypotheses testing: Hierarchical linear modeling

In the HLM analyses, a fully unconditional, intercept-only model for NA, IWB, and KNS was first estimated to examine supervisor within-group and between-group variability. Significant within-group variances were found (NA: σ^2^ = 0.206, *p* < 0.001; IWB: σ^2^ = 0.188, *p* < 0.001; KNS: σ^2^ = 0.073, *p* < 0.001). Significant between-supervisor variances in groups were also found (NA: τ = 0.859, *p* < 0.001; IWB: τ = 1.006, *p* < 0.001; KNS: τ = 1.005, *p* < 0.001).

The intra-class correlation coefficient for NA, IWB, and KNS was 0.806, 0.842, and 0.932, respectively, above the threshold of.05 (Heck et al., 2014). In other words, 80.6% of the total variation in NA, 84.2% of the total variation in IWB, and 93.2% of the total variation in KNS occurs between supervisor groups. The significant between- and within-group variances indicate that there may be supervisor-related factors that help to explain variation between supervisors in NA, IWB, and KNS. In other words, these variances demonstrate the nested nature of data and justify our use of multi-level analyses. The differences in Chi-square tests with deviance values indicated that Model 2 represented a significantly better fit than Model 1 (NA: Δχ^2^(1) = −50.139, *p* < 0.001; IWB: Δχ^2^(1) = −30.224, *p* < 0.01; KNS: Δχ^2^(1) = −32.756, *p* < 0.001) and Model 3 had a better fit than Model 2 (NA: Δχ^2^(1) = −19.885, *p* < 0.001). Pseudo *R*^2^ values were 0.063, 0.055, and 0.110 for NA, IWB, and KNS, respectively, supporting the validity of all models. Table 3 presents the results of examining the causal and moderation hypotheses (i.e., H1, H2a, H2b, H4, and H5) using HLM.

**TABLE 3:** Results of causal and moderation analysis using HLM.

Predictor	Negative affectivity (NA)			Innovative work behavior (IWB)		Knowledge sharing (KNS)	
	Model 1	Model 2	Model 3	Model 1	Model 2	Model 1	Model 2
**Step 0: No variable**							
Intercept	2.227***	0.914***	1.677**	3.646***	4.519***	3.426***	4.173***
	(0.086)	(0.231)	(0.554)	(0.092)	(0.168)	(0.088)	(0.140)
**Step 1: Independent variables**							
CCB		0.399***H1	0.089				
		**(0.061)**	(0.223)				
PL		0.152***	−0.262				
		(0.042)	(0.106)				
Gender		−0.214	0.306				
		(0.101)	(0.300)				
NA					−0.393***H2a		−0.337***H2b
					**(0.066)**		**(0.053)**
**Step 2: Interaction terms**							
PL * CCB			0.156***H4				
			**(0.037)**				
Gender * CCB			−**0.122_H5_**				
			**(0.122)**				
**Model fit**							
σ2	0.206***	0.220***	0.216***	0.188***	0.199***	0.073***	0.082***
τ	0.859***	0.508***	0.426***	1.006***	0.739***	1.005***	0.723***
ρ	0.806	0.698	0.663	0.842	0.788	0.932	0.898
Deviance	519.033	468.894	449.009	529.543	499.319	452.267	419.511
ΔDeviance		−50.139***	−19.885***		−30.224***		−32.756***
Pseudo *R*^2^		0.063	0.018		0.055		0.110

*N* = 137(supervisors); *N* = 207 (employees); The regression coefficients are the unstandardized coefficients from HLM; Values in parentheses display the standard error from HLM; *p < 0.05. **p < 0.01, and ***p < 0.001 (two-tailed); *σ*^2^ = Variance within groups (*σ*^2^_w_); τ = Variance between groups (*σ*^2^_B_); ρ = Intra-class correlation coefficient (ICC); Deviance = -2 × log-likelihood of the full maximum-likelihood estimate (is a measure of model fit; the smaller it is, the better the model fits); CCB, compulsory citizenship behavior; PL, passive leadership; NA, negative affectivity; IWB, innovative work Behavior; KNS, knowledge sharing; H1, evidence to support H1^+^; H2, evidence to support H2^–^; H3, evidence to support H3^–^; H6, evidence to support H6^+^; H7, evidence to reject H7. Bold Numerics represent the significant/important results of that specific model.

**FIGURE 2:**
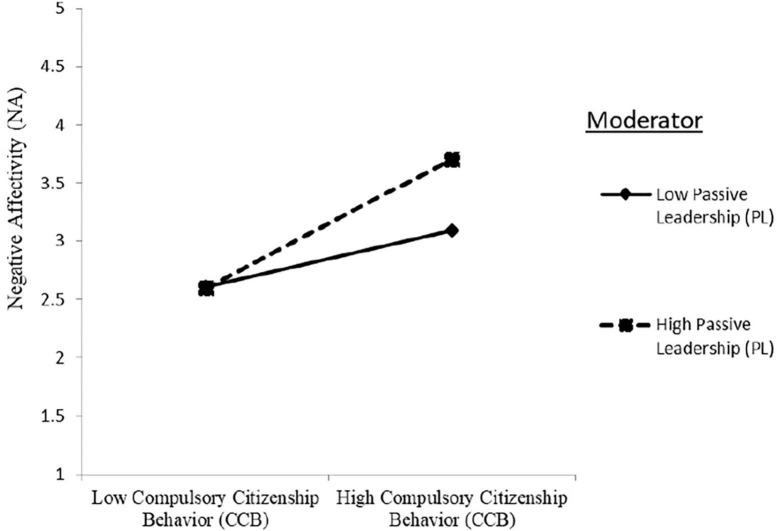
Moderation effect of passive leadership on the relationship between compulsory citizenship behavior and innovative affectivity.

Table 3 shows, CCB had a significant positive effect on NA (NA: Model 2 in Table 3: γ = 0.399, *p* < 0.001), while NA significantly negatively predicted IWB (IWB: Model 2 in Table 3: γ = -0.393, *p* < 0.001) and KNS (KNS: Model 2 in Table 3: γ = -0.337, *p* < 0.001), providing support for H1^+^, H2a^–^, and H2b^–^, respectively. The interaction term of CCB × PL in predicting NA was significantly positive (NA: Model 3 in Table 3: γ = 0.156, *p* < 0.001), providing support for H4^–^. The interaction term of CCB × Gender in predicting NA was not significant (NA: Model 3 in Table 3: γ = −0.122, *p* > 0.05). Therefore, H5 is rejected. The plotted interaction in Figure 2 unveiled that CCB increased NA to a higher degree when PL was high rather than low, which confirmed H4 regarding the positive moderating role that PL plays in the positive relationship between CCB and NA.

### 5.3. Hypotheses finding: Hayes’s PROCESS

Table 4 presents the results of examining the mediation (i.e., H3a and H3b) and moderated-mediation hypotheses (i.e., H6a, H6b, H6c, and H6d) using Hayes’s PROCESS.

**TABLE 4:** Results of mediation and moderated-mediation analyses using PROCESS V3.5 by Andrew F. Hayes.

Model	Standardized effect (β)	SE	LL95%CI	UL95%CI
**Mediation model (model 4 of Hayes PROCESS macro)**				
**Bootstrapping results for indirect effect of CCB on IWB *via* NA**				
CCB→NA→IWB	−0.300* H3a	0.052	-0.404	-0.198
**Bootstrapping results for indirect effect of CCB on KNS *via* NA**				
CCB→NA→KNS	−0.316*H3b	0.055	-0.424	-0.207
**Moderated-mediation model (model 10 of Hayes PROCESS macro)**				
**Bootstrapping results for conditional indirect effect of CCB on IWB *via* NA at different levels of PL and gender**				
Low PL (-1SD = −1.210) and Female (-1SD = −0.430)	-0.155	0.103	-0.361	0.047
Low PL (-1SD = −1.210) and Male (+ 1SD = −0.570)	-0.003	0.119	-0.212	0.251
Medium PL (0SD = 0.000) and Female (-1SD = −0.430)	-0.320*	0.070	-0.458	-0.187
Medium PL (0SD = 0.000) and Male (+ 1SD = −0.570)	-0.168	0.102	-0.352	0.046
High PL (-1SD = 0.1.210) and Female (-1SD = −0.430)	-0.485*	0.062	-0.604	-0.362
High PL (-1SD = 1.210) and Male (+ 1SD = −0.570)	-0.333*	0.106	-0.534	-0.118
Index of moderated-mediation effect of PL	-0.136*	0.040	-0.219	-0.064
Index of moderated-mediation effect of gender	0.152	0.115	-0.058	0.391
**Bootstrapping results for conditional indirect effect of CCB on KNS *via* NA at different levels of PL and gender**				
Low PL (-1SD = −1.210) and Female (-1SD = −0.430)	-0.149	0.100	-0.345	0.053
Low PL (-1SD = −1.210) and Male (+ 1SD = −0.570)	-0.003	0.116	-0.201	0.251
Medium PL (0SD = 0.000) and Female (-1SD = −0.430)	-0.308*	0.068	-0.446	-0.178
Medium PL (0SD = 0.000) and Male (+ 1SD = −0.570)	-0.162	0.102	-0.352	0.045
High PL (-1SD = 0.1.210) and Female (-1SD = −0.430)	-0.467*	0.062	-0.586	-0.348
High PL (-1SD = 1.210) and Male (+ 1SD = −0.570)	-0.320*	0.108	-0.537	-0.113
Index of Moderated-Mediation effect of PL	-0.131*	0.039	-0.214	-0.061
Index of moderated-mediation effect of gender	0.147	0.114	-0.069	0.380

*N* = 137 (supervisors); *N* = 207 (employees); SE = standard error; *p < 0.05, **p < 0.01, and ***p < 0.001 (two-tailed); CCB, compulsory citizenship behavior; PL, passive leadership; NA, negative affectivity; IWB, innovative work behavior; KNS, knowledge sharing; H4, evidence to support H4; H5, evidence to support H5; H8, evidence to support H8^+^; H9, evidence to support H9^+^; H10, evidence to reject H10^+^; H11, evidence to reject H11^+^.

The results indicate NA mediates the negative effect of CCB on IWB and KNS. Therefore, H3a and H3b were both supported. Additionally, the bootstrapping estimation on the indirect effect of CCB on IWB through NA was negative and significant [β = −0.300, the 95% CI using a 5,000 bootstrap sample does not include 0: CI (−0.404, −0.198)]. Similarly, the indirect effect of CCB on KNS through NA was negative and significant [β = -0.316, 95% CI (−0.424, −0.207)].

H6a and H6c propose that stronger PL and females would strengthen the indirect negative relationship between CCB and IWB through NA. Bootstrapping results indicate that the conditional indirect effect is negatively significant and strong for males in the high PL condition [β = −0.333; 95% CI (−0.534, −0.118)], negatively significant and strong for females in the high PL condition [β = −0.485; 95% CI (−0.604, −0.362)], negatively insignificant and moderate for males in the medium PL condition [β = −0.168; 95% CI (−0.352, 0.046)], negatively significant and moderate for females in the medium PL condition [β = −0.320; 95% CI (−0.458, −0.187)], negatively insignificant and weak for males in the low PL condition [β = −0.003; 95% CI (−0.212, 0.251)], and negatively insignificant and weak for females in the low PL condition [β = −0.155; 95% CI (−0.361, 0.047)].

Furthermore, the indices of moderated mediation, which test the difference between a high, medium, and low level of PL between males and females as multiple conditional effects, show that the six multiple conditional effects significantly differ from each other for the moderated-mediation effect of PL [Index = −0.136, SE = 0.040, 95% CI (−0.219, −0.064)], but they insignificantly differ from each other for the moderated-mediation effect of gender [Index = 0.152, SE = 0.115, 95% CI (−0.058, 0.391)]. Therefore, H6a is supported, while H6c is rejected.

H6b and H6d propose that stronger PL and females would strengthen the indirect negative relationship between CCB and KNS through NA. Bootstrapping results indicate that the conditional indirect effect is negatively significant and strong for males in the high PL condition [β = −0.320; 95% CI (−0.537, −0.113)], negatively significant and strong for females in the high PL condition [β = −0.467; 95% CI (−0.586, −0.348)], negatively insignificant and moderate for males in the medium PL condition [β = −0.162; 95% CI (−0.352, 0.045)], negatively significant and moderate for females in the medium PL condition [β = −0.308; 95% CI (−0.446, −0.178)], negatively insignificant and weak for males in the low PL condition [β = −0.003; 95% CI (−0.201, 0.251)], and negatively insignificant and weak for females in the low PL condition [β = −0.149; 95% CI (−0.345, 0.053)].

**FIGURE 3:**
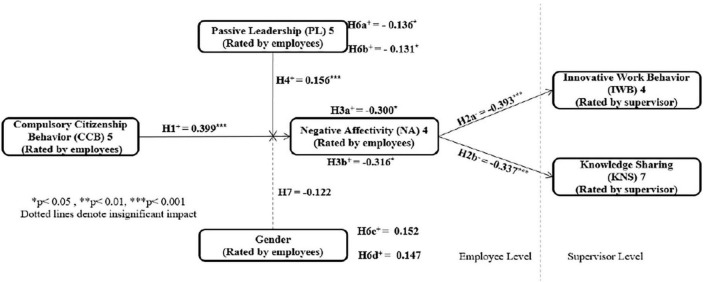
Model of findings and estimation results. *p < 0.05, **p < 0.01, and ***p < 0.01. Dotted lines denote insignificant impact.

Furthermore, the indices of moderated mediation, which tests the difference between a high, medium, and low level of PL between males and females as multiple conditional effects, show that the six multiple conditional effects significantly differ from each other for the moderated-mediation effect of PL [Index = −0.131, SE = 0.039, 95% CI (−0.214, −0.061)], but they insignificantly differ from each other for the moderated-mediation effect of gender [Index = 0.147, SE = 0.114, 95% CI (−0.069, 0.380)]. Therefore, H6b is supported while H6d is rejected. Figure 3 represents the model of findings and the results of examining research hypotheses.

## 6. Discussion

While responding to the call for further attention to the boundary conditions and potential psychological mechanisms linking workplace stressors to employee IWB and creativity (He et al., 2020), we postulated and tested a moderated-mediation model on the relationship between CCB and employee IWB and KNS. Results revealed that CCB is negatively related to NA. It is also evident from the results that NA partially mediates the negative relationships between CCB and employee IWB and KNS. Similarly, results indicate that PL moderates the positive relationship between CCB and NA. Lastly, it was also found that the indirect negative relationship between CCB and employee IWB and KNS through NA is moderated by PL. As expected, the findings of this study are much in line with the results of previous studies focusing on the relationship between workplace stressors, including hindrance stressors, abusive supervision, employee creativity, and work–family conflict (Kwan et al., 2018; Zhang et al., 2018; He et al., 2020; Chen et al., 2022).

Our study confirms the role of CCB as a hindrance stressor that elicits negative emotions in employees, who ultimately withhold their IWB and show their reluctance to share their tacit and explicit knowledge with their colleagues. This particular attitude of employees can be fatal for organizations in today’s competitive environment in which there is a greater need for innovation and KNS. This study supports the notion that the hazardous effects of CCB are stronger than the positive effect of OCB (He et al., 2020). Findings of this study revealed that CCB has a direct impact on NA, thereby establishing that today’s aggravated workplace demands pressure employees to reluctantly exhibit extra-role behavior (Vigoda-Gadot, 2006), which later on overwhelms individuals with tasks beyond their ability and reach. Contending with COR theory and the findings of Tang and Vandenberghe (2021), this study argues that CCB disempowers employees of their physical, cognitive, and emotional resources as they invest them in the additional informal tasks conferred on them due to CCB. This over-exhaustion of resources deprives and drains individuals of the requisite resources required for formal pending tasks, inevitably leading to higher NA, which in turn transforms into the withdrawal of IWB and KNS. The significance of this study for theory development and practice is discussed below.

### 6.1. Theoretical implications

This study is a pioneering attempt to explore the negative effect of CCB and has several valuable contributions to understanding the relationship between CCB and employee IWB and KNS behaviors. First, using the COR theory framework, this study extends the research on the consequences of CCB for employees and organizations. Current research on CCB mainly focuses on the concept and measurement of CCB (Vigoda-Gadot, 2006), the similarity and differences between CCB and OCB (Ahmadian et al., 2017), and the predictors of CCB such as destructive leadership, neuroticism, and abusive supervision (Zhao et al., 2014; Youn et al., 2017; Wu et al., 2018). However, in the past two decades, a few researchers, such as Tepper et al. (2004), Vigoda-Gadot (2006, 2007), and Zhao et al. (2014), have paid attention to the “dark side” of OCB and have focused more on the consequences of CCB. Nevertheless, these studies mainly focused on employee emotions, psychology, and attitudes and paid less attention to employee performance and behaviors. A few recent studies have been conducted to identify the impact of CCB on employee behaviors, such as employee creativity (He et al., 2020) and work–family conflict (Chen et al., 2022). This research is still in its infancy, particularly the link between CCB and employee IWB and KNS behaviors through the mediating lens of NA. Therefore, by focusing on employee IWB and their KNS behaviors, this study strives to uncover the dark side of CCB through the mediating lens of NA.

Second, we believe the moderating effects of PL on the relationships between CCB and NA can be understood in terms of COR theory. Consistent with COR theory, we suggest that high CCB works as a stressor for employees in that it represents a threat to resource loss/gain. We argue that passive leaders, who are highly unconcerned with the benefits of their employees/organization in the context of high CCB, will not serve as an important resource to employees in this context. Consistent with our pattern of results, we suggest that subordinates of passive leaders are more likely to withhold their efforts toward their job, colleagues, and organization. As a result, the negative effect of CCB on employee NA is enhanced in the presence of a passive leader and will ultimately transform into the withdrawal of IWB and KNS.

Third, this study also extends the existing body of employee IWB and KNS behaviors literature by identifying its new antecedents, such as CCB. As we know the importance of innovation and KNS behaviors in an organization, research on these topics is attracting considerable attention of researchers. Previous studies have identified two main stressors: hindrance stressors and challenge stressors. Research is still in its infancy in identifying how these two types of stressors affect employee IWB and KNS behaviors. Apart from the traditional workplace stressors (i.e., job insecurity, time pressure), this study has considered a non-traditional workplace stressor (CCB) and attempted to explore its impact on employee IWB and KNS behaviors.

### 6.2. Managerial implications

This study has been an attempt to understand the structural relationships between CCB, leadership, affectivity, and performance outputs, providing a glimpse as to how academics experience, respond to, and deal with non-traditional/unconventional workplace stressors. It also extends our knowledge of the factors of IWB and KNS in an educational setting, offering several contributions to the current body of knowledge for the future development of related literature. This research offers several worthwhile insights into the ways in which NA can be managed for academics in higher education institutions, thereby mitigating the likelihood of negative approaches to and behavior at work.

First, this study proposes that the compulsory extra-role behaviors that are undertaken against academics’ will (i.e., CCB) (Vigoda-Gadot, 2007) do exist in higher education institutions and have a negative impact on academics’ IWB and KNS. Supervisors should be aware that citizenship conduct has negative consequences, along with the normal positive gains. While an academic’s spontaneous manifestation of extra-role behavior, in addition to in-role behavior, has become an important index for measuring the university’s effectiveness, it is also costly. Engaging in intensive compulsory behavior is a fundamental force that leaves academics nervous and fatigued by feeling highly pressured and thus consumes the cognitive resources required to generate novel ideas. Hence, if supervisors want to improve employees’ IWB and stimulate KNS, academics should not only be encouraged to collect needed knowledge but also enhance the work requirements and working resources so that academics’ affective state is high. In that sense, academics will be supported to embrace challenging aspects of their work and to apply efforts in improving performance, which in turn induces creativity and donating knowledge to others. Supervisors can stimulate a high affective state by creating a positive work environment that controls and reduces CCB by deploying adequate intervention strategies. The institution must protect and compensate “excellent academics” (i.e., those who exhibit CB) by rewarding their extra-role performance properly.

Second, the mediating role of NA serves as a critical context through which CCB can produce a deterring effect on academics’ performance output that supervisors strive to nurture. Given that CCB practices are sometimes unavoidable, supervisors must implement effective supportive practices and assist academics in regulating their emotions in order to experience better work sentiments and avoid further degeneration into more unfavorable results. Specifically, supervisors should design training programs to support academics in handling and processing their feelings and emotions, followed by promoting past successful innovation experiences and KNS practices. In that sense, regulating NA through better coping strategies with difficult emotions can help employees to better regulate their emotions upon encountering pressure associated with experiencing extra-role behaviors. Simultaneously, training modules, as well as additional support in the form of increased work resources and organizational supports (e.g., implicit innovation information sharing, supportive leadership, trust, timely feedback, and so on), can assist academics in pursuing unique and creative activities.

Third, the current study demonstrates the crucial role of passive leaders in amplifying the detrimental effects of negative emotions on academics’ performance outputs as a result of CCB.

Further to the above, CCB is unlikely to be completely eliminated from the workplace; therefore, ensuring that assigned tasks are perceived as purposeful and meaningful requires supervisors in institutions to take an active role in curbing the deleterious effect of the PL style by ensuring that their supervisors avoid PL qualities and practices. In light of the current situation, academic institutions should place serious efforts into fostering active and positive leadership styles among their supervisors, such as nurturing transformational leaders (Barling and Frone, 2017), and devise strategies and plans in the face of PL practices, such as better managerial selection. In that sense, institutions must provide leadership counseling and training to effectively identify and resolve PL, with the goal of eventually implementing 360-degree feedback.

## 7. Conclusion, limitations, and future research

This study develops a moderated-mediated model based in an attempt to advance the literature on extra-role performance behaviors among academics, particularly innovative work behaviors and knowledge sharing, through the lens of work stressors. Results show that academics’ compulsory citizenship behaviors positively influence negative affectivity, which, in turn, negatively impacts academics’ innovative work behavior and knowledge sharing. The detrimental effect of compulsory citizenship behaviors on negative affectivity is then positively moderated by passive leadership, which amplifies this relationship. The combined effect of compulsory citizenship behaviors and negative affectivity exerted on innovative work behavior and knowledge sharing are magnified amid the elevated presence of passive leadership, while gender does not significantly influence this association.

Despite the fact that our study has yielded useful outcomes, there are still some limitations that need to be addressed. First, data were collected from academics in the UAE higher education sector, and, hence, data generalizability to a larger population in other countries is limited. As a result, replicative studies might utilize the same set of key variables and the study’s structural model to address other countries with different types of institutions (such as state universities or community colleges). Second, this study explored the relationship between three primary variables–CCB, NA, and performance output–over a lengthy period while recognizing that these constructs can fluctuate in a matter of days. As a result, in the future, scholars may utilize additional qualitative methodologies such as diary studies to further support the examination of the structural model. In this sense, experiences of CCB by faculty at different time intervals might have varying negative effects on performance outputs over time. Finally, this study unveils the moderating role of PL, which might strengthen the deleterious effect of CCB conduct on performance outputs. Further research could address different moderating variables that could strengthen or weaken this relationship, such as characteristics of leaders based on trait theory of leadership, despotic leaders, or the leader–member exchange leadership style, and the dark and bright sides of academics’ personality traits in relation to emotion regulation and delusions.

## Data availability statement

The raw data supporting the conclusions of this article will be made available by the authors, without undue reservation.

## Ethics statement

Ethical review and approval was not required for the study on human participants in accordance with the local legislation and institutional requirements. The patients/participants provided their written informed consent to participate in this study.

## Author contributions

RA and NS contributed to the conception and design of this study and organized the database. RA performed the statistical analysis. MK and UR wrote the first draft of the manuscript and wrote the sections of this manuscript. All authors contributed to the manuscript revision, proofread, and approved the submitted version.
